# How to Enter the Medical Profession

**Published:** 1886-12-11

**Authors:** 


### How to Enter the Medical Profession.

W. H. B. writes to ask "how a young man of limited means" may best enter the medical profession, and requests us to give him "an idea of the probable expense." It is not unlikely that a good many young men and their parents are in some uncertainty on the same subject, and we shall therefore try to give as much useful information as possible in a limited space. Everybody remembers Punch's "advice to those about to marry"—"*don't*," said Mr. Punch. We will not exactly say to those desiring to enter the medical profession, "don't," but we will say, "don't do it without giving the matter the fullest consideration. Think, in the first place, whether you have natural aptitudes for careful and continuous study; and then think whether your resources are sufficient to enable you to pursue your studies to their legitimate end without strain to your mind or health. And, finally, think what available means you may have for "getting into practice" after you have obtained the necessary qualifications."

Practical, if not Palatable.

The *learned professions*, as they are somewhat incorrectly called—that is to say, the Church, Physic, and the Law—look enviable from the outside: but they do not present quite the same aspect from within. A brave appearance requires brave means: and *thousands* of poor clergymen, lawyers, and doctors would tell you they would prefer the brave means to the brave appearance *now*, although in their earlier days the appearance had most attraction for them. It is a pity—a very great pity—that handicrafts are so despised among us. They are healthy, they are honest, and they are as much worthy of respect, when conscientiously pursued, as any other calling whatever. Medicine is an honest calling—or at least may be made so; but it is not very healthy, and it is decidedly not profitable. The initial outlay and the long waiting for results are not adequately rewarded in the end. To use a homely proverb, "The game is very seldom worth the candle." This is carefully considered advice by "one who knows." If, however, in spite of warning, you still incline, like the Scotch couple who insisted upon being married, although the minister told them he knew "marriage to be a blessing to few, a curse to many, and a trouble to all,"—if, we say, you still incline to knock at the gates of the profession, and even to force your way in—why, then, so be it, but you must take the consequences.

The Best and Cheapest Way.

Which is the best way in—and the cheapest? The cheapest is not the best, nor the best the cheapest. If a few words be said about *all* the ways, the aspirant can then take his choice. "To be or not to be" M.D.? That is one of the first questions. The M.D. degree, however it be obtained in the British Isles, is more costly in point of money, and more exacting in point of labour and time, than the licentiateships and memberships of the Colleges of Physicians and Surgeons. The entrance examinations are more extensive and more severe; the professional curricula are also considerably more extensive and laborious. This is absolutely true, whatever any inconsiderate person may say to the contrary, and can easily be proved when it is necessary. The cheapest and easiest ways of getting into the medical profession are by the halls and colleges, not by the universities. The most costly, but the best ways, are by the universities. The following is, approximately, the order of cheapness in halls, colleges, and universities, with the minimum of cost in each case. The expenditure includes class fees, books, clothes, and living for three or four years.

| Qualification. | Average Term of Study. | Minimum Cost. |
|---|---|---|
| | Years. | |
| L.S.A. London .. .. | 3 to 4 | £350 to 400 |
| L.F.P.S. Glasgow .. .. | 3 „ 4 | 350 „ 400 |
| L.K.Q.C.P. Ireland .. | 3 „ 4 | 350 „ 400 |
| L.R.C.S. Edinburgh .. | 4 | 400 „ 450 |
| L.R.C.P. Edinburgh .. | 4 | 400 „ 450 |
| M.R.C.S. England .. | 4 | 400 „ 450 |
| L.R.C.P. London .. .. | 4 | 400 „ 450 |
| M.D. Aberdeen .. .. | 4 to 5 | 500 „ |
| M.D. Glasgow .. .. | 4 „ 5 | 500 „ 550 |
| M.D. Edinburgh .. .. | 5 | 600 „ 700 |
| M.D. London .. .. | 5 to 6 | 700 „ 800 |
| M.D. Cambridge .. .. | 7 „ 8 | 800 „ 1000 |
| M.D. Oxford .. .. | 7 „ 8 | 800 „ 1000 |

University Degrees.

In the case of Oxford and Cambridge every candidate must take his B.A. degree before or during his term of medical study, and that is the reason why the whole period of university life is two or three years longer than at other universities. The actual period of purely medical studies is the same for Oxford and Cambridge as for other M.D. degrees. In addition to the above there are three other degree-conferring bodies in Great Britain, and several in Ireland; but as these latter are seldom sought by English or Scotch students, they are not named in detail. The British ones are — the Victoria University, Manchester, and the Universities of Durham and St. Andrews. The status of their degrees, and probably the cost of obtaining them, are, for various reasons, somewhat lower than those of other universities. From a social point of view the degrees of Oxford and Cambridge stand highest. From a scientific and medical standpoint those of London and Edinburgh are at the head.

Places of Study.

With regard to places of study, London and Edinburgh offer the greatest advantages; but the other Scotch Universities, and also several provincial towns in England where there are large hospitals, give complete courses. The Victoria University, Manchester, gives a full qualifying curriculum, and the degrees of Bachelor and Doctor of Medicine, at a reasonable cost. The amounts here given are for necessary expenditure AFTER the passing of the preliminary examination in Arts, and registration as a medical student. If more detailed information be required on any particular point, it will be given in answer to questions in future numbers of THE HOSPITAL.

